# Mirror Neuron System and Upper-Limb EMG Activity During Reaching Imitation in Stroke Survivors: Comparing Outcomes After Observing Normal vs. Aberrant Movements

**DOI:** 10.1177/10538135251407110

**Published:** 2026-01-22

**Authors:** A Sulfikar Ali, Ashokan Arumugam, Mayur Bhat, Hari Prakash Palaniswamy, Selvam Ramachandran, Senthil Kumaran D

**Affiliations:** 1 Department of Physiotherapy, Kasturba Medical College Mangalore, Manipal Academy of Higher Education, Manipal, India; 2 Department of Physiotherapy, College of Health Sciences, University of Sharjah, Sharjah; United Arab Emirates; 3 Sustainable Engineering Asset Management Research Group, RISE-Research Institute of Sciences and Engineering, University of Sharjah, Sharjah, United Arab Emirates; 4 Department of Physiotherapy, Manipal College of Health Professions, Manipal Academy of Higher Education, Manipal, India; 5 Neuromusculoskeletal Rehabilitation Research Group, RIMHS—Research Institute of Medical and Health Sciences, University of Sharjah, Sharjah, United Arab Emirates; 6 Department of Audiology and Speech Language Pathology, Kasturba Medical College Mangalore, Manipal Academy of Higher Education, Manipal, India; 7 Department of Speech and Hearing, Manipal College of Health Professions, Manipal Academy of Higher Education, Manipal, India

**Keywords:** electromyography, mirror neuron activation, movement error, movement observation, Mu suppression, upper extremity

## Abstract

**Purpose:**

To assess how brain cortical activity and upper limb (UL) muscle activity associated with the imitation of a UL reaching task differ following action observation of normal and aberrant movement conditions.

**Materials and Methods:**

In this cross-sectional study, 17 individuals who had unilateral stroke were asked to watch a UL reaching task performed with normal and aberrant movement patterns shown with prerecorded videos and then imitate normal movement patterns. Electroencephalographic mu-rhythm activity, a measure of the mirror neuron system (MNS), and the electromyographic amplitudes of four paretic UL muscles (percentage maximum voluntary contraction) were measured during action observation and imitation (AOI) of normal and aberrant conditions. Freidman's ANOVA was used to compare the outcomes across the conditions.

**Results:**

EEG analysis revealed statistically significant suppression of mu-rhythm (demonstrating better MNS activity) during the AOI of normal movement than during aberrant movement conditions at the C3 (p = 0.001) and C4 (p = 0.003) electrodes. Furthermore, the amplitude of percentage maximum voluntary contraction for the supraspinatus muscle significantly increased (p = 0.027) during imitation of the task following observation of the normal movement condition.

**Conclusion:**

AOI of normal movements resulted in better MNS activity and increased supraspinatus muscle activity than did the observation of aberrant movements. These findings support the incorporation of therapist-guided AOI training focused on normal movement patterns and the avoidance of exposure to aberrant models as a low-cost, neurophysiology-driven adjunct in stroke rehabilitation protocols.

**Trial Registration:**

Clinical Trials Registry-India (CTRI) identifier: CTRI/2018/04/013466.

## Introduction

Stroke ranks as the third most common cause of disability and the second highest cause of death worldwide (Johnson et al., 2016). The loss of upper limb (UL) function is the most prevalent debilitating motor deficit that occurs after a stroke (Sale et al., 2014). The prevalence of UL impairment in the acute phase of stroke is approximately 50–80%, and that in the chronic phase is 40–50% (Hussain et al., 2018). Epidemiological studies have shown that 60% of persons with partial or complete impairment of UL function cannot perform activities normally up to six months after stroke, which accounts for a poor functional prognosis (Kwakkel et al., 2003; van Kuijk et al., 2009). Therefore, UL rehabilitation is essential for improving activities of daily living (ADL) in people with stroke (L. Borges et al., 2018).

Empirical evidence supports the use of action observation and imitation (AOI)-based interventions for functional training of the UL in patients with stroke (L. Borges et al., 2018; Cowles et al., 2013; Kim, 2015; Sale et al., 2014). AOI is a physical rehabilitation approach that facilitates the occurrence of neural plasticity through the activation of the mirror neural system (MNS), promoting motor recovery in people with stroke (L. R. Borges et al., 2022). AOI serves as a priming method to enhance motor cortex excitability prior to or in conjunction with rehabilitation (Choi et al., 2022; Stoykov et al., 2015). In AOI, an individual is asked to observe a task executed by another healthy person through a video (Calvo-Merino et al., 2005) or a real-time demonstration (Cowles et al., 2013) and a subsequent imitation of the same task, which will activate the same group of neurons that is responsible for the observed action (L. R. Borges et al., 2022; Garrison et al., 2013; Sarasso et al., 2015). The MNS, which is found particularly in the parietal and frontal cortices, is activated as a result of this process, which is founded on the mechanism of neuroplasticity (Garrison et al., 2013; Patel, 2017). AOI is an important foundation for understanding and learning actions through mimicking (Mukamel et al., 2010; Rizzolatti & Sinigaglia, 2016) and executing the observed behavior (Ertelt et al., 2007). Experimental evidence has shown that the cortical motor areas are stimulated both when performing the task and when mentally practicing or observing the action of interest (Jeannerod, 2001).

Motor neuron activation during AOI occurs in a way similar to that of action execution by creating an internal picture of the action and thereby leading to motor (re)learning (Marangon et al., 2014). This is attributed to the activation of the MNS, which is most often assessed by electroencephalography (EEG) (Frenkel-Toledo et al., 2014; Hofree et al., 2015; Zhang et al., 2018). Simultaneously, the impact of this activation on the neuromuscular system can be evaluated peripherally by assessing muscle activity via electromyography (EMG) (Hobson & Bishop, 2016; Hofree et al., 2015). The key indicator of MNS activity is the suppression of mu rhythm (Fox et al., 2016; Hobson & Bishop, 2016). Mu is a spectrum of EEG oscillations ranging from 8 to 13 Hz within the alpha band and is recorded from the sensorimotor cortex of the brain (Amoruso & Urgesi, 2016; Frenkel-Toledo et al., 2014). On the other hand, measuring muscle activity via EMG enables us to understand peripheral motor system function associated with the central motor cortex (Hofree et al., 2015).

AOI intervention for the UL has widely incorporated demonstration of a reaching task performed by a normal individual to the target population. Studies have examined various contexts (such as the social context (Donne et al., 2011), visual context (Amoruso & Urgesi, 2016; Iacoboni et al., 2005), etc.), and the context of interest has been demonstrated to alter brain activity (Riach et al., 2018). Therefore, the movement conditions presented in the video during AOI may influence stroke rehabilitation. In healthy individuals, observing normal hand movements appears to produce greater somatosensory activation and MNS activity than observing aberrant or clumsy movements (Cheng, 2018). In clinical settings, individuals with stroke may observe their own affected limb or observe other patients in the rehabilitation ward performing tasks with compensatory strategies that are considered abnormal (Cheng, 2018). Exposure to such aberrant movement patterns may unintentionally reinforce maladaptive strategies and interfere with optimal motor learning.

Moreover, observing the affected limb performing a task can provide feedback about the errors or abnormal components in the movement, helping patients recognize and correct abnormal components to move toward more normal patterns. Previous studies have indicated that error-driven learning processes enhance central adaptability and motor skill development and that error augmentation can promote greater changes in performance (Israely & Carmeli, 2016; Seidler et al., 2013; Wei et al., 2005). Thus, error-enhancing approaches may support functional motor recovery following brain injury (Wei et al., 2005).

Therefore, it remains unclear whether AOI should emphasize normal movements or aberrant movements to maximize rehabilitation outcomes, which needs further substantiation. In the present study, our objective was to investigate whether imitation of a UL reaching task following the observation of aberrant movement patterns influences MNS activation (mu suppression via EEG) and UL muscle activity (via EMG) better than following the observation of normal movement patterns in people with stroke.

## Materials and Methods

### Study Design and Setting

A cross-sectional study was conducted at Kasturba Hospital in Manipal, India, specifically within the Departments of Physiotherapy and Speech-Language Pathology. This study was reported in accordance with the STROBE checklist (Vandenbroucke et al., 2007). The institutional ethics and research committees (IEC: 66/2018) of Kasturba Medical College and Kasturba Hospital, Manipal, granted their approval prior to the start of the study. Additionally, the study has been registered with the Clinical Trial Registry-India under the registration number CTRI/2018/04/013466.

### Study Participants

A purposive sampling method was used to identify post-stroke survivors aged 18 to 80 years who were diagnosed with unilateral stroke and had a Brunnstrom voluntary control grade (Shah et al., 1986) of ≥2 to ≤6 in the paretic UL, a Montreal Cognitive Assessment score (MoCA) (Nasreddine et al., 2005) of ≥26 and an intact ability to imitate score (Decety et al., 1997) of ≥8 with a non-paretic UL. We excluded persons who had (1) visual impairment; (2) global or receptive aphasia; (3) perceptual deficits such as unilateral neglect, anosognosia, apraxia, and difficulty with right/left discrimination; (4) a history of seizures and/or taking medications for epilepsy; (5) any other medical problems or comorbidities that precluded their participation in the study; and (6) any contraindications for performing an EEG. The study procedures were explained in detail to the identified participants, and informed consent was obtained from all individuals before the start of the study.

### Procedure and Experimental Setup

**Figure 1.**
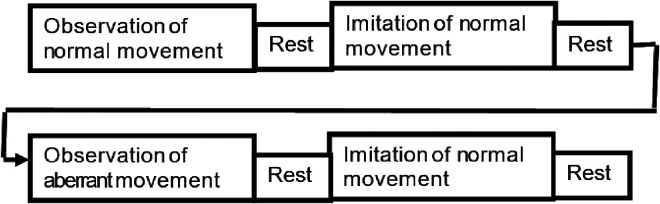
Box Car Paradigm Representing the Study Procedure. The Paradigm Consists of an Observation of Normal and Aberrant Movement Patterns Followed by an Imitation of Normal Movement. A Rest Period of 2 min was Given Between Each Block.

**Figure 2.**
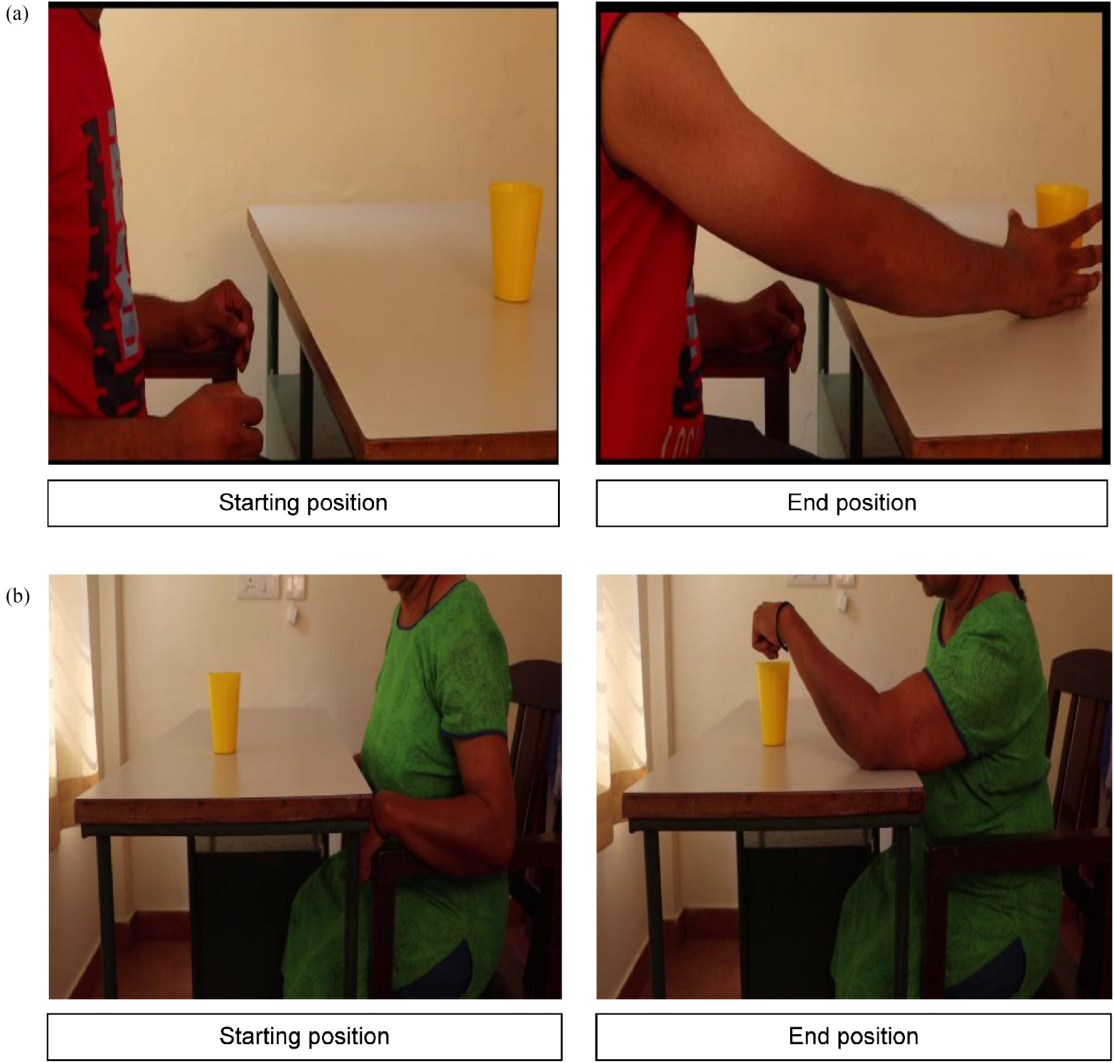
Illustration of Two Different Conditions of an Upper Limb Reaching Task: **(a)** Starting and Ending Positions of a Reaching Task Performed with a Normal Movement Pattern by a Healthy Individual Without Any Abnormal Compensatory Movement Patterns; **(b)** Starting and Ending Positions of a Reaching Task Performed with Aberrant (Compensatory Synergistic) Movement Patterns by a Participant Post-Stroke. In Both Conditions, the Study Participants Were Asked to Observe the pre-Recorded Videos from a Participant Perspective. The Task Consisted of Reaching and Holding a Glass Kept at a Distance of One Arm's Length on a Table.

All the participants were assessed by one of the investigators pursuing a Master in Physiotherapy (Neurosciences) for inclusion criteria using the Brunnstrom stages of recovery (for the UL) (Shah et al., 1986), intent to imitate scale (Decety et al., 1997), and MOCA scale (Nasreddine et al., 2005). Each participant was instructed to relax and sit comfortably in a chair. They were instructed to place their affected UL on the arm of the chair, with the shoulder abducted to 10°, the elbow bent at 90°, the forearm fully pronated, and the wrist in a neutral position. All the participants received a brief overview of the EEG and surface EMG procedures. The sequence of the AOI intervention is illustrated via a “boxcar paradigm” (see Figure 1). Participants were asked to observe two pre-recorded videos (each lasting for 2 min) of a UL reaching task from their own perspective. The videos were presented to each participant in the following sequence:(1) a UL reaching task performed by a healthy individual with normal movement patterns (Figure 2a) and (2) a UL reaching task performed by a person with a stroke with aberrant movement patterns (Figure 2b). Then, the participant was asked to imitate the task repeatedly for 2 min using normal movement patterns shown in video 1 irrespective of observing video 1 (Figure 2a) or 2 (Figure 2b). A rest period of 2 min was given between each block to avoid the carry-over effects with the previous videos. EEG mu rhythm suppression and EMG muscle activity (percentage maximum voluntary contraction [%MVC]) were recorded during AOI of the task following observation of normal and aberrant movement conditions. The Template for Intervention Description and Replication (TIDieR) checklist was utilized to report the intervention details (Supplementary material 1) (Hoffmann et al., 2014).

#### EEG Setup

**Figure 3.**
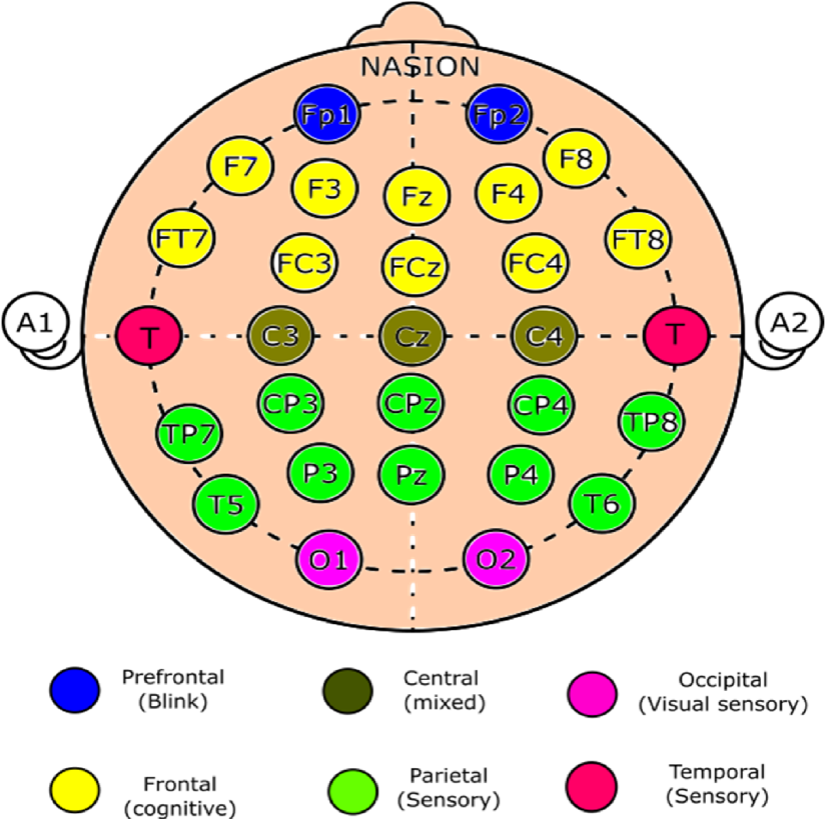
Electrode Placement for EEG. Electrode Placement via a 32-Channel Electrode Cap According to the 10–20 Classification. The Electrodes are Color-Coded, and the Regions of Interest are Specified.

To prepare for EEG recording, the electrode locations on the scalp were treated with a skin-preparing gel (Quik Cell and Nuprep). A 32-channel electrode cap was positioned on each participant's scalp, with EEG electrodes arranged according to the international 10–20 system and reference electrodes placed bilaterally over the mastoid processes (Figure 3). To increase the conductivity, a conduction paste (Quik Cell Electrolyte and Ten-20) was applied. Each electrode's impedance was maintained below 5 kΩ, whereas the interelectrode impedance was maintained within 2 kΩ. The EEG recordings were gathered via the Compumedics Neuroscan 4.5 systems™ (Neuroscan Laboratories, Australia) and processed with Neuroscan 4.5 software.

#### EMG Setup

**Figure 4.**
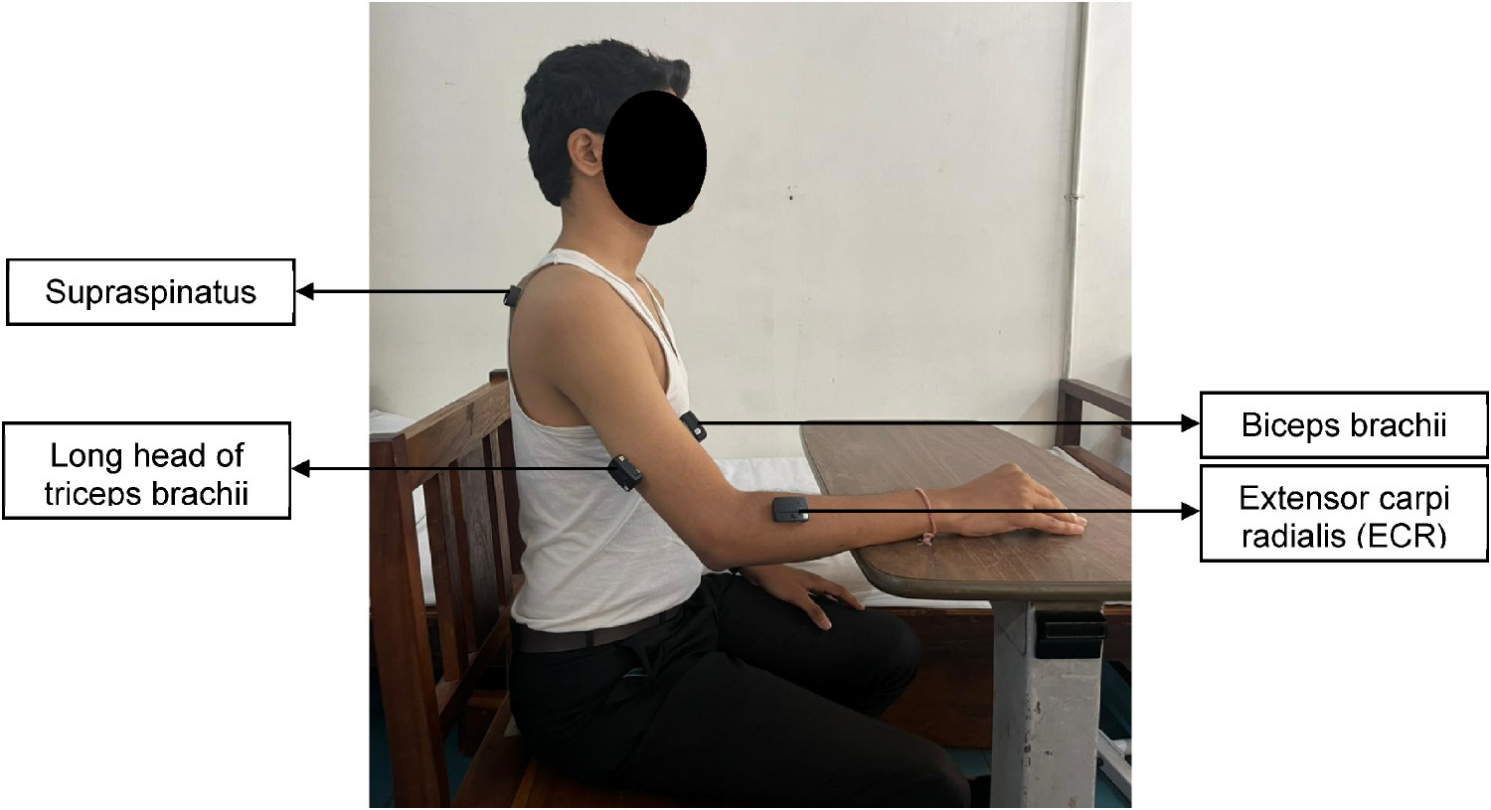
Electrode Placements for EMG. Placement of EMG Electrodes Over Supraspinatus, Biceps Brachii, Long Head of the Triceps Brachii, and Extensor Carpi Radialis (ECR) Muscles to Record the Muscle Activity.

The muscle activity of four muscles of the paretic UL (the supraspinatus, biceps brachii, long head of the triceps brachii, and extensor carpi radialis [ECR]) was captured via an 8-channel Delsys, Trigno wireless EMG system (AD Instruments, USA, model number—DSY-DS-T01D-4, 2016) (Lee et al., 2015) (Figure 4). These four muscles were selected because they function as primary movers or stabilizers of the UL during forward-reaching movements, consistent with previous studies investigating UL muscle activity during reaching tasks using EMG (Lee et al., 2015; Tokuda et al., 2016; Wagner et al., 2007). The skin overlying the muscles of interest was cleaned with a sterilium (propanol). Wireless EMG sensors were secured with adhesive tape over the muscle bulk in a parallel orientation as per the recommendations of the Surface Electromyography for the Non-Invasive Assessment of Muscles (SENIAM) guidelines.

### Data Acquisition and Processing

#### EEG Data

Raw EEG data were sampled at 1000 Hz, and MATLAB was used for analysis. Prior to interpolating the EEG channels via the spherical spline approach, the continuous EEG data were examined visually for any missing information through the BESA 4 shell dip fit spherical model. The data were bandpass filtered with an online analogue filter between 1 and 100 Hz and then smoothed via a low-pass filter of 30 Hz. By epoching the raw EEG files with a 0–500 ms time window relative to stimulus onset, the files were transformed into average files. Using the “runica” command of the EEG Lab, independent component analysis (ICA) breaks down multivariate event-related potential waveforms into their source subcomponents. Multiple artifact rejection algorithms (MARAs) (Winkler et al., 2011) were then used to analyse the ICA-processed waveforms. These algorithms automatically exclude components that include artifacts on a number of factors. The waveforms are referred to as a common average. MATLAB code was used for averaging and re-referencing. The two central electrodes, C3 and C4, covering the sensorimotor cortex were used to calculate the absolute power of the mu frequency bands. We chose locations C3 and C4 close to the sensory‒motor region, which are frequently documented in action observation studies (Frenkel-Toledo et al., 2014; Muthukumaraswamy & Johnson, 2004; Pfurtscheller et al., 2006) and are utilized to record mu rhythm activity (Hobson & Bishop, 2016). The derived data (in .txt format) were transferred to a Microsoft Excel (Redmond, Washington, USA) document for statistical analysis.

#### EMG Data

Delsys electromyography devices were used to preamplify the EMG signals (0–1.5 mv) after they were recorded at 1000 Hz. Using Labchart software (version 8.1.13; Labchart, AD Instruments, Australia; model number: MLS060/8), the wireless EMG signals were amplified (x1000), bandpass filtered (20–500 Hz), and sampled at 4 kHz. Then, they were analysed via the root mean square method for 20 ms epochs. For the supraspinatus, biceps brachii, triceps brachii, and ECR, the EMG amplitudes recorded during movement imitation were normalized and are presented as a percentage of the maximal voluntary contraction (%MVC).

## Statistical Analysis

The sample size calculation was conducted via G*Power version 3.1 (Faul et al., 2007) on the basis of a repeated-measures ANOVA (within-subject factors) method, which was considered an appropriate analysis for the planned within-subject comparisons. The parameters used were as follows: test family = *F* tests, effect size = 0.25, α = 0.05, power (1−β) = 0.80, four measurements (normal vs. aberrant movement observation and imitation), assumed correlation among repeated measures = 0.7, and nonsphericity correction = 0.8. The analysis indicated that a minimum of 17 participants would be required to achieve sufficient power for the intended comparisons. Although the final statistical analyses employed nonparametric tests (Friedman and Wilcoxon) due to data nonnormality, the a priori estimation using a parametric model was retained as a conservative and widely accepted approximation, given the limited availability of validated nonparametric power analysis methods for repeated-measures designs (Faul et al., 2007; Lehmann, 1975).

Statistical analysis was performed via the Statistical Package for the Social Sciences Version 16.0 (SPSS Inc., Chicago, III, USA 2007). The normality of the data distribution was examined via the Shapiro‒Wilk test. The data from the C3 and C4 electrodes (EEG data) were analysed separately during the observation and imitation of the normal and aberrant movement conditions via 2 × 2 Friedman's ANOVA because the data were skewed. The %MVC EMG amplitudes and mu suppression scores were compared across test conditions via Wilcoxon signed rank tests with Bonferroni adjustments. Median differences between test conditions (aberrant movement condition—normal movement condition) were calculated for both the normalized EMG amplitudes (%MVC) and the mu suppression score.

## Results

**Figure 5.**
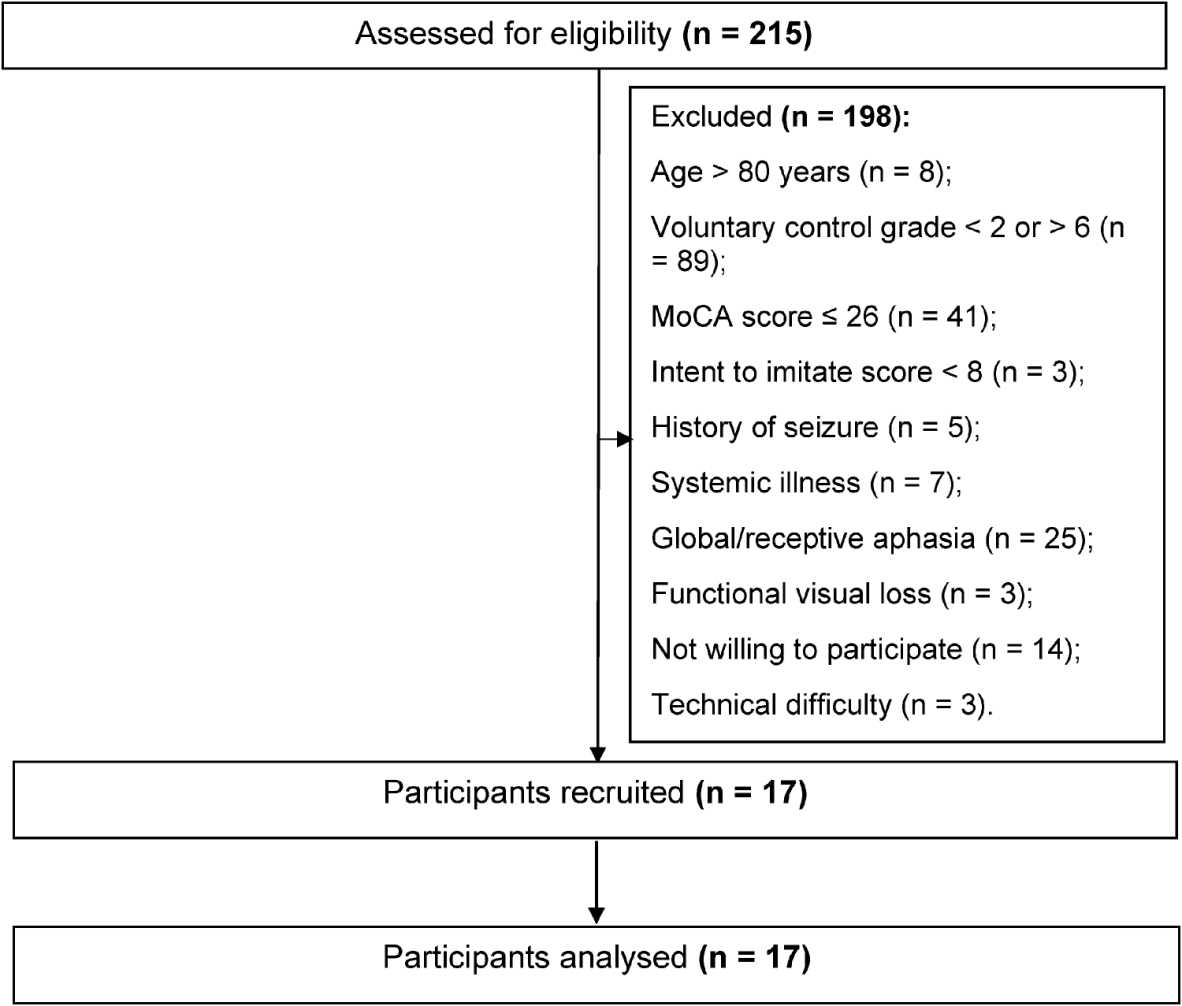
Flow of Participants in the Study.

For the study, 215 stroke survivors were screened. Among them, 198 did not meet the inclusion criteria. Figure 5 represents the participant flow from screening to final analysis. Seventeen participants (13 men and 4 women; aged 54.29 ± 16.07 years) were recruited for the study and completed the analysis.

The participants’ clinical characteristics and demographics are displayed in Table 1. The majority of the participants (n = 11) had a history of ischemic stroke, with a mean (SD) Fugl-Meyer assessment-upper extremity (FMA-UE) motor score of 35.41 (16.42). Sixteen participants presented with elbow flexor spasticity of varying scores on the modified Ashworth scale ranging from 1–3. Five participants had spasticity scores ranging from 1 to 2 for wrist flexors (Table 1).

**Table 1.** Participant Characteristics (n = 17).

Variables	Descriptive statistics
Age in years	54.29 ± 16.07^b^
Gender (Male: Female)	13(76%):4(24%)^a^
Side of hemiparesis (Right: Left)	10(58%):7(42%)^a^
Type of stroke (Ischemic: Hemorrhagic)	11(64%):6(36%)^a^
Poststroke duration in days	24 (8.00,185.0)^c^
Fugl-Meyer score – Upper limb: Motor	35.41 ± 16.42^b^
Voluntary control stage	
Upper limb	3 (3,4)^c^
Hand	3 (3,4)^c^
MOCA score	29.59 ± 1.00^b^
Intent to imitate score	10 (10,10)^c^
MAS Score	
Elbow flexors (n = 17)	
Grade 0	1 (5.8%)^a^
Grade 1	11 (64.7%)^a^
Grade 1+	2 (11.7%)^a^
Grade 2	2 (11.7%)^a^
Grade 3	1 (5.8%)^a^
Wrist flexors (n = 17)	
Grade 0	12 (70.5%)^a^
Grade 1	1 (5.8%)^a^
Grade 2	4 (23.5%)^a^

a = Number (percentage), b = Mean ± standard deviation, c = Median (Q1, Q3); MAS- Modified Ashworth scale; MOCA- Montreal Cognitive Assessment.

### EEG Analysis

**Figure 6.**
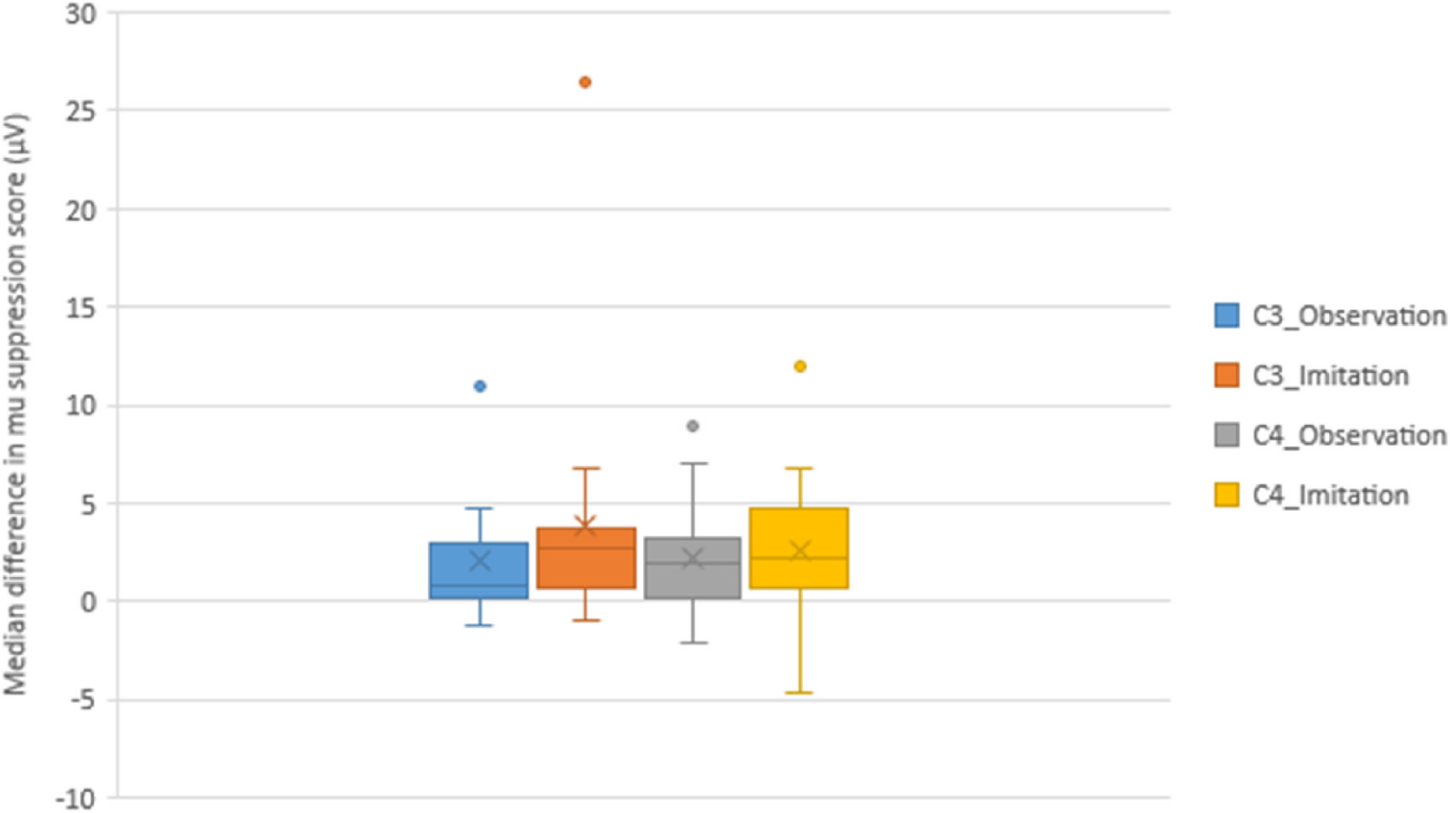
The Median Difference and Interquartile Range of mu Suppression Scores (μV) in the C3 and C4 Areas Showing Differences Between Test Conditions (Normal vs. Aberrant Movement Conditions) During the AOI of a UL Reaching Task.

During the AOI of the UL reaching task, Friedman's ANOVA demonstrated a significant difference in mu suppression between the normal and aberrant movement conditions at the C3 (p = 0.001) and C4 (p = 0.003) electrodes. Significant suppression of mu rhythm (indicating greater MNS activation) was observed during the observation of the normal movement condition than during the aberrant movement condition at both the C3 and C4 electrodes, according to the post hoc analysis of mu rhythm scores via the Wilcoxon signed rank test with Bonferroni adjustments (p = 0.011) (Table 2). The median differences in mu suppression scores between test conditions (normal vs. aberrant movement conditions) in the C3 (observation [0.87], imitation [2.68]) and C4 areas (observation [1.98], imitation [2.20]) are depicted in Figure 6.

**Table 2.** Comparison of the mu Suppression Score (μV) Across Test Conditions (Normal and Aberrant Movement Patterns) in the C3 and C4 Areas (n = 17).

EEG Electrode placement Areas	Friedman's ANOVA	AOI Task	Movement Conditions		Median difference (Q1, Q3)^a^	p value
			Normal movement median (Q1, Q3)	Aberrant movement median (Q1, Q3)		
C3	**0**.**001**	Observation	1.23 (0.41,2.36)	2.41 (1.16, 4.66)	0.87 (0.23,2.95)	**< .001** ^b^
		Imitation (of normal movement)	0.99 (0.45,1.90)	4.17 (2.13, 6.62)	2.68 (0.69,3.75)	**< .001** ^b^
C4	**0**.**003**	Observation	1.33 (0.39, 2.83)	2.74 (0.77,7.40)	1.98 (0.12,3.17)	**0.001** ^b^
		Imitation (of normal movement)	1.15 (0.62, 2.19)	3.69 (2.78, 5.43)	2.20 (0.72,4.77)	**0.011** ^b^

^a^ Median difference = aberrant movement condition—normal movement condition.

^b^ Significant p values based on the Wilcoxon signed rank tests with Bonferroni adjustments.

### EMG Analysis

**Figure 7.**
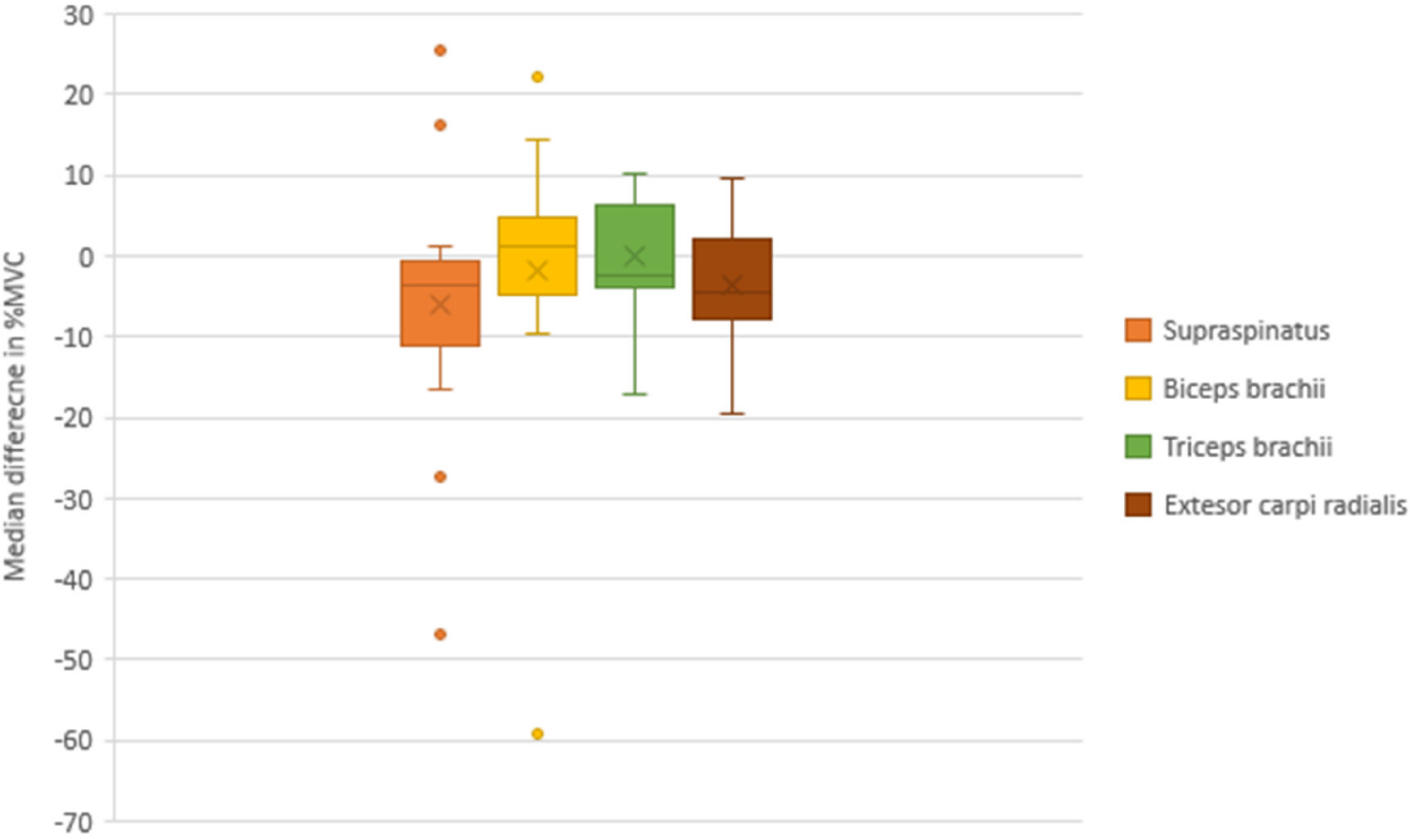
Median Difference and Interquartile Range of Normalized Electromyographic Amplitudes (% Maximum Voluntary Contraction [%MVC]) of the Supraspinatus, Biceps Brachii, Triceps Brachii and ECR Showing Differences Between Test Conditions (Normal vs. Aberrant Movement Conditions) During AOI of a UL Reaching Task.

Analysis of the %MVC of paretic UL muscles with the Wilcoxon signed rank test revealed a significant increase in supraspinatus muscle activity (p = 0.027), but not for the other three muscles, during imitation of the reaching task following observation of the normal movement condition compared with the aberrant movement condition (Table 3). The median differences in normalized EMG amplitudes (%MVC) between test conditions (normal vs. aberrant movement conditions) in supraspinatus (−3.49), biceps brachii (1.20), triceps brachii (−2.27) and ECR (−4.37) activity are depicted in Figure 7.

**Table 3.** Comparison of Normalized Electromyographic Amplitudes (%MVC) Between Test Conditions (Normal and Aberrant Movement Conditions) (n = 17).

	Movement conditions		Median difference (Q1, Q3)^a^	p value
	Imitation (of normal movement) following observation of normal movement—median (Q1, Q3)	Imitation (of normal movement) following observation of aberrant movement—median (Q1, Q3)		
Supraspinatus	52.19 (40.87, 57.13)	46.45 (31.88, 51.95)	−3.49 (−9.64, −1.21)	**0**.**027**^b^
Biceps Brachii	50.51 (43.64, 59.88)	50.45 (43.17, 63.37)	1.20 (−4.08, 4.55)	1.000
Triceps Brachii	72.54 (65.04, 79.42)	72.95 (62.02, 82.62)	−2.27 (−3.97, 5.32)	0.678
Extensor Carpi Radialis (ECR)	69.42 (66.59, 74.65)	65.49 (61.75, 75.54)	−4.37 (−5.47, −1.61)	0.159

^a^ Median difference = aberrant movement condition—normal movement condition

^b^ Significant p values based on the Wilcoxon signed rank tests with Bonferroni adjustments

## Discussion

The study highlights the unique design in which action observation is delivered with an imitation of normal movement patterns soon after observation of two different conditions (normal and aberrant movement). The literature has reported that learning from errors is a basic principle of motor skill acquisition (Seidler et al., 2013), and error-enhancing training could be a useful strategy for helping people with brain injuries restore their functional motor skills (Wei et al., 2005). On this basis, we hypothesized that observing aberrant movements would provide feedback about errors encountered during the performance of movement to the participant, thereby refining motor control and improving performance. This helps the participants obtain better ideas about the error components to be eliminated while performing the reaching task by detecting discrepancies between intended and actual movements. However, we could not observe the beneficial effect of the AOI of aberrant movement patterns on the mirror neuron system or muscle activation.

The suppression of mu rhythm, a reliable electrophysiological marker of MNS activation (Frenkel-Toledo et al., 2014; Gazzola et al., 2007), observed during the AOI of normal movement conditions could be explained by various factors. First, the normal movement condition is simple and familiar compared with the aberrant movement condition, which is relatively novel, complex, and unfamiliar to the participants post-stroke (Caspers et al., 2010; Cheng et al., 2017; Gazzola et al., 2007; Sun et al., 2016), When the participants were asked to observe an aberrant movement condition and imitate the normal movement condition, the anticipated motor plan could have been disrupted or incongruent with the observed action, which would have compromised MNS activation (Caspers et al., 2010; Cheng et al., 2017). Our findings are consistent with those of earlier research on healthy subjects (Cheng, 2018; Cheng et al., 2017). When the median nerve was stimulated and cortical activity was measured via magnetoencephalography (MEG) in healthy individuals, the motor cortex showed greater activation and improved functional connectivity within the somatosensory system during the observation of normal hand movements as opposed to abnormal movements (Cheng, 2018; Cheng et al., 2017). Another study reported that when correct goal-directed hand movements were observed, there was more sensorimotor activation, as indicated by event-related desynchronization of the alpha and beta bands (Wriessnegger et al., 2013). Studies that have assessed the effects of congruent and incongruent videos on visuomotor integration via event-related potentials and functional magnetic resonance imaging (fMRI) have revealed decreased brain activity during the AOI of incongruent videos (Sim et al., 2015). Although we have not analysed the activity of the visual cortex during AOI of normal and aberrant movement conditions, we believe that the observation of normal videos stimulated the ventral and dorsal visual cortex and would have facilitated visual object recognition and visuomotor integration. These results, along with our findings, suggest that observing normal and congruent actions induces stronger motor cortical activities and promotes better functional connectivity in the somatosensory system. Second, the AOI of tasks with a greater level of motor control, such as normal movement conditions, could have led to higher activity in the MNS than in the aberrant movement conditions, which exhibit a synergetic, distorted pattern (Cheng et al., 2017). Third, the primary motor cortex is more activated when goal-directed movement is observed than when non-goal-directed movement is observed since motor cortex activity increases when participants observe normal and goal-directed UL movements and produce better motor learning processes (Caspers et al., 2010; Cheng et al., 2017; Deconinck et al., 2015; Sun et al., 2016). A meta-analysis by Caspers et al. also suggested that goal-directed actions lead to stronger activation of the MNS (Caspers et al., 2010).

This study also aimed to observe whether the central cortical changes observed through EEG are translated into the peripheral motor system. After normal movement conditions were observed, the EMG amplitude of the supraspinatus muscle increased significantly, whereas the EMG amplitudes of the biceps brachii, triceps brachii, and ECR muscles increased non-significantly during task imitation. The proximal musculature is hierarchically activated first to drive movement at the distal component in space, which may account for the higher muscular activity in the supraspinatus relative to the other muscles (McCrea et al., 2005). According to preliminary data, there may be a connection between MNS activity and an increase in supraspinatus activity when mimicking the UL-reaching task after normal movements are observed. The structural connections between the MNS and the motor cortex could be the reason for this (Cerri et al., 2003; Shimazu et al., 2004). Earlier studies have demonstrated strong relationships between the primary motor cortex and the MNS (more especially, the premotor region F5) (Iacoboni et al., 1999; Shimazu et al., 2004). Motor responses to action observation may depend on the attention of the participant. Therefore, it is possible that the participants focused on the goal and pattern of the UL movement condition, which could have influenced muscle activity during the AOI of the normal movement condition (Hetu et al., 2010).

It is possible that the spasticity of the elbow and wrist flexors is the reason why the EMG amplitudes of the other three muscles are not significant (Subramanian et al., 2018). In addition, during observation of an aberrant movement, the central nervous system could have realized abnormal events or synergies (shoulder abduction, elbow flexion, and wrist flexion) as a part of error detection and allowed reduced muscle activity in the UL muscles while performing the task. However, further studies are warranted to substantiate this hypothesis. Since this was a cross-sectional study, the aberrant movement was shown only once, and repeated practice of the task is likely required for motor (re)learning to occur. Following a stroke, motor (re)learning involves a complicated progression of cognitive, associative, and autonomous phases as well as a change from declarative to procedural knowledge. This could explain why we did not observe a beneficial effect of action observation on aberrant movement patterns. Future research should investigate whether repetitive AOI of aberrant movements over a period of time leads to changes in cortical brain activity and peripheral muscle activation.

According to our study, one of the important contexts that might influence AOI was the condition (normal vs aberrant movement condition) in which the action was presented for observation (Gazzola et al., 2007). This finding adds to the current evidence that movement conditions (normal/aberrant) may also influence MNS activity. However, further neurophysiological or hemodynamic studies are warranted to disentangle the underlying mechanisms post-stroke. We believe that optimized motor learning and motor control can be promoted by the observation of correct, normal movement and that the congruency of movement conditions contributes to facilitating corticospinal excitability during AOI. The present findings have potential implications for designing structured AOI protocols in clinical rehabilitation settings. The observed cortical and muscular activation patterns during the observation of normal movements suggest that repetitive exposure to correctly executed actions, under therapist guidance, may help reinforce appropriate motor representations and facilitate motor relearning in stroke patients. Integrating such structured AOI sessions alongside task-oriented training could enhance motor recovery by promoting cortical reorganization and improving movement quality. Furthermore, potential strategies to minimize patients’ exposure to aberrant-movement observations in a real-world context include offering tablets or screens with preselected videos of correct task performance rather than relying on casual observation of the ward; using group-based therapy sessions in which patients are grouped at the functional level so that individuals exhibiting severe compensatory movements are not observed by those in earlier stages of recovery; incorporating mental practice of normal movement patterns; creating designated treatment zones or visual barriers in busy wards so that patients are shielded from constant exposure to others’ compensatory movements; and using digital tools that present normal movement models for home-based observation, thus reducing reliance on watching aberrant movements. Additionally, patients are informed about what constitutes aberrant movements and advised that if they observe such movements, they should not imitate them. This approach aids in showing how a movement should not be performed, thereby facilitating error-augmentation and error-driven learning.

Our finding of greater mu rhythm suppression at C3 and C4 during the AOI of normal movements aligns with the broader neurophysiological literature, which has demonstrated that mu desynchronization reflects the activation of motor simulation networks. Multimodal studies combining EEG with fMRI have shown that mu-suppression during action observation corresponds with increased blood oxygen level-dependent (BOLD) responses in motor and premotor cortices, supporting its validity as a marker of mirror-neuron system engagement (Arnstein et al., 2011; Yin et al., 2016). Although simultaneous EEG–fMRI work in AOI remains limited, these findings reinforce the translational importance of using EEG-derived mu-suppression to indicate cortical activation during AOI paradigms. Such approaches are particularly valuable in low-resource settings where access to fMRI may be limited.

Although this cross-sectional design allowed us to differentiate the cortical and muscular responses to normal versus aberrant movement observation, it does not permit conclusions about long-term therapeutic effects. Future research employing a pre–post intervention design or randomized controlled trial design are warranted to determine whether the observed neural and muscular changes translate into functional gains and support the therapeutic use of AOI in stroke rehabilitation.

## Methodological Considerations and Recommendations

This study has some methodological issues that need to be taken into account before the findings can be interpreted. First, even though the tests were conducted in a controlled setting, the lengthy procedure made it impossible to eliminate participant distraction completely. Nonetheless, the data obtained were both technically good and suitable for analysis. Second, we might have overlooked analysing the MNS observed in the premotor and parietal cortices since we only examined the central C3 and C4 electrodes of the EEG. fMRI studies to determine cortical area activation during normal or aberrant movement observation and imitation are warranted in people with stroke to reaffirm the findings of our study. In addition, we did not obtain any verbal responses from the participants to understand whether visual observation of normal movement helps them improve UL function compared with aberrant movement observation. Future studies should consider the behavioral feedback of patients as well as changes in their movement trajectory after observing normal movements. The relatively small sample size and the use of Bonferroni correction to control multiple comparisons may have increased the likelihood of type II errors. Future studies with larger sample sizes are warranted to increase the statistical sensitivity and to validate the observed trends. Another potential limitation of this study is that most participants exhibited mild to moderate spasticity in the elbow flexors. As a result, the EMG signals obtained may partially reflect altered muscle tone or impaired neuromuscular activation associated with spasticity rather than purely voluntary muscle activity. This factor may limit the generalizability of our EMG findings to stroke populations without spasticity. Nevertheless, we included these participants to preserve the sample size and ensure that our findings represent a clinically realistic poststroke population.

## Conclusion

AOI demonstrated an increase in MNS activity and normalized EMG activity in the supraspinatus following the observation of normal movements compared with aberrant movements. Therefore, to improve UL function in stroke patients, it is important to observe normal movements when structuring the AOI treatment module. The therapeutic effects of current stroke rehabilitation programs might be enhanced by the incorporation of this observational component of everyday activities.

## Supplemental Material

sj-docx-1-nre-10.1177_10538135251407110 - Supplemental material for Mirror Neuron System and Upper-Limb EMG Activity During Reaching Imitation in Stroke Survivors: Comparing Outcomes After Observing Normal vs. Aberrant Movements Supplemental material, sj-docx-1-nre-10.1177_10538135251407110 for Mirror Neuron System and Upper-Limb EMG Activity During Reaching Imitation in Stroke Survivors: Comparing Outcomes After Observing Normal vs. Aberrant Movements by A Sulfikar Ali, Ashokan Arumugam, Mayur Bhat, Hari Prakash Palaniswamy, Selvam Ramachandran and Senthil Kumaran D in NeuroRehabilitation
