# Supplementary material for: Mirror Neuron System and Upper-Limb EMG Activity During Reaching Imitation in Stroke Survivors: Comparing Outcomes After Observing Normal vs. Aberrant Movements
Source: NeuroRehabilitation. 2026 Jan 22;58(2):200–13. doi: 10.1177/10538135251407110 (PMC12963472; doi:10.1177/10538135251407110)
Supplement: sj-docx-1-nre-10.1177_10538135251407110 - Supplemental material for Mirror Neuron System and Upper-Limb EMG Activity During Reaching Imitation in Stroke Survivors: Comparing Outcomes After Observing Normal vs. Aberrant Movements [file sj-docx-1-nre-10.1177_10538135251407110.docx]

**Supplementary materials**

**Supplementary material 1.** Template for intervention description and replication (TIDieR) checklist for reporting of intervention

| TIDieR Items | Description of items |
| --- | --- |
| 1. Name of the intervention | AOI of a UL reaching task. |
| 2. Rationale | AOI has been considered an important basis for understanding and learning an action by mimicking (Mukamel et al., 2010; Rizzolatti & Sinigaglia, 2016) and consecutively executing the observed action (Ertelt et al., 2007). It is based on the mechanism of neuroplasticity, leading to the activation of MNS present in the speciﬁc areas of the brain, thereby improving the UL function following stroke (Garrison et al., 2013; Patel, 2017). |
| 3. Materials used in the intervention | A chair with an armrest, a plastic glass, a computer, a table of height (~29.5”), and pre-recorded videos of reaching tasks for AOI. |
| 4. Intervention procedures | Participants were made to sit in front of a table kept 2 inches apart from the arm of the chair. A cylindrical shaped plastic glass was kept on the table at one arm's distance for each participant based on their arm length to elicit complete elbow extension. Participants were asked to observe two pre-recorded videos (each lasting for 2 min) of a UL reaching task from their own perspective. The videos were presented to each participant in the following sequence:(1) a UL reaching task performed by a healthy individual with normal movement patterns and (2) a UL reaching task performed by a person with a stroke with aberrant movement patterns. Aberrant movements refer to movements performed by person with a stroke using their affected limbs during a reaching task, exhibiting a compensatory synergetic pattern. Aberrant movement videos were created for each individual, as the movement characteristics during a reaching task vary from person to person. Both normal and aberrant movement videos were recorded from four different perspectives: anterior, posterior, lateral, and superior. The arm involved in the action was isolated from any unwanted elements in the scene, such as the actor or background. Then, the participant was asked to imitate the task repeatedly for 2 min using normal movement patterns shown in video 1 irrespective of observing video 1 (Figure 2a) or 2 (Figure 2b). A rest period of 2 min was given between each block to avoid the carry-over effects with the previous videos. Participants were provided with videos demonstrating either normal movements of the right or left arm, depending on the side involved, to enhance understanding of the required movement. Verbal cues were given to the participant during imitation of the normal movement conditions. EEG mu rhythm suppression and EMG muscle activity (percentage maximum voluntary contraction [%MVC]) were recorded during AOI of the task following observation of normal and aberrant movement conditions. |
| 5. Provider | The intervention was provided by a neurological physiotherapist (SA) qualified and trained in the rehabilitation of people with stroke. |
| 6. Mode of intervention  delivery | Individual therapy sessions. |
| 7. Setting of intervention | Study screening, interventions, and assessments were conducted at the physical therapy department of a tertiary hospital located in India. |
| 8. Dosage | This intervention was a single therapy session consisting of observation and imitation of two movement conditions. Video of each movement condition lasted for 2 min for action observation, followed by imitation of the task for 2 min. A rest period of 2 min was given between each task. The total duration of intervention was 18-20 min. |
| 9. Tailoring | Nil |
| 10. Modifications: | The intervention was not modified during the course of the study. |
| 11. Fidelity assessment | Intervention was supervised by a neurological physiotherapist (SK) with 20 years of experience in the rehabilitation of people with neurological disorders. |

*AOI, action observation and imitation; MNS, mirror neuron system; UL, upper limb*
